# Application of gradient-consensus Richardson–Lucy deconvolution to noisy undersampled brightfield microscopy data

**DOI:** 10.1364/BOE.597710

**Published:** 2026-05-15

**Authors:** Yiming Liu, Sjoerd Stallinga

**Affiliations:** Department of Imaging Physics, Delft University of Technology, Delft, The Netherlands

## Abstract

The gradient-consensus Richardson–Lucy (GC-RL) deconvolution algorithm is a novel approach to contrast restoration in high-resolution microscopy imaging without excessive noise amplification. We evaluate this method in this work, focusing on the impact of the noise level of the input image, of imperfections and deviations in image formation from the ideal incoherent case, and of using undersampled input data. The gain in spectral signal to noise ratio (SSNR) is larger when the noise level of the raw image is lower. We also find a positive impact on SSNR gain from using an optical transfer function (OTF) in the deconvolution that has a decreased spatial frequency response compared to the ideal incoherent OTF. A simple upsampling scheme, embedded in the GC-RL deconvolution, is demonstrated to be suitable for undersampled data. We show our results on simulated data, on fluorescence microscopy data, and on brightfield microscopy data of a breast tumour section. The GC-RL method has potential for whole slide imaging technology to retrieve diagnostically relevant image features at the smallest length scales from scanned images at lower resolution, providing a gain in throughput and data storage requirements.

## 1. Introduction

Optical microscopy is essential for biological studies and pathology diagnoses, as it provides information on the cellular and tissue scale. The resolution, however, is fundamentally constrained by the diffraction limit. In addition, optical aberrations and camera readout and shot noise reduce contrast and information content of the acquired image. Computational techniques such as deconvolution have therefore been developed to enhance contrast and image quality [1]. Among all existing deconvolution methods, the Richardson–Lucy (RL) algorithm remains one of the most widely used algorithms because of its simplicity and the guaranteed positivity of the deconvolution outcome [2,3]. The algorithm is a form of Maximum Likelihood Estimation (MLE) given the Poissonian nature of the photon statistics of the image signal. The main drawbacks of the RL-deconvolution algorithm are its lack of convergence and noise amplification [4]. Recently, we demonstrated a connection between the ill-convergence and the noise amplification of RL-deconvolution using a Crámer Rao Lower Bound (CRLB) analysis [5].

In the past few decades several methods have been proposed to address these issues [6–13]. A regularization approach based on the L1 norm or L2 norm has been proposed to constrain the noise amplification [6,7]. The balance between noise suppression and reconstruction of object details, however, is hard to determine when using these additional norms. Several alternative forms of the RL update rule have been developed from a purely mathematical point of view to provide faster convergence speed while keeping a relatively good level of stability [8]. Several researchers have developed different reference maps to extract edge regions for better edge sharpening while reducing the ringing effect [9–11]. Even though this can result in a better extraction of high frequency details above the noise level, all these maps only focus on edge information and therefore the noise amplification effect is not suitably taken into account. Another advance has been the proposal of a three-level hierarchical frequency separation approach that performs independent deconvolution within each band to suppress noise and ringing [12]. The drawback of this method is that it requires setting multiple ad-hoc parameters, making it quite cumbersome to configure for optimum outcome.

Recently, the Gradient-Consensus Richardson–Lucy (GC-RL) algorithm was proposed to overcome the limitations of the RL-deconvolution algorithm [14]. The local update step in each iteration is no longer uniformly applied, but only if consistency with the underlying data warrants it. This is achieved by random binomial data splitting [15] of the acquired image in two noise independent data halves and checking the consistency between the two local updates of the two independent data halves. Only in case of consistency does the overall deconvolution estimate receive an update. In this way, the iterative deconvolution is effectively locally stopped if the algorithm only tries to fit the local noise pattern. In this way a fair balance between enhancement and noise is achieved. Its performance in practical microscopy applications, however, has not been investigated in depth.

In particular, automated microscopy slide scanning for digital pathology applications, so-called Whole Slide Imaging (WSI), poses opportunities and challenges for applying deconvolution algorithms. WSI platforms make 2D scans of ∼ 4 µm thick stained tissue slices mounted on a brightfield microscope slide [16–19]. State-of-the-art systems make scans of typically 15mm×15mm areas at a sampling distance of ∼ 0.25 µm within a minute, usually with a 20×/NA0.75 objective. This results in Gigapixel colour (RGB) images with several Gigabyte (lossless compressed) file sizes. The highest resolution (smallest sampling distance) is used to identify features such as mitotic figures, irregularly shaped cell nuclei indicating cells caught in the action of division. These are a few µm in size, and are often used in cancer diagnostics [20,21]. Most routine diagnostic applications, however, need an overview of tissue structure, for which a lower resolution (larger sampling distance) suffices. This suggests an opportunity for applying deconvolution in WSI setting. Scanning tissue slides with e.g. a 10×/NA0.45 objective combined with contrast and resolution retrieval with deconvolution on areas of interest could reduce the amount of produced data fourfold, while still presenting a pathologist with the diagnostically relevant detail on the smallest length scale.

It is the goal of this work to assess the potential of GC-RL deconvolution to address this opportunity. Several challenges have to be addressed to this end. The first challenge deals with noise robustness. In addition to the shot noise, inherent to any optical imaging technique, camera systems used in WSI practice also introduce readout noise. This raises the question how much readout noise can be tolerated for successful application of GC-RL deconvolution. The second challenge concerns the underlying image formation. Brightfield microscopes are partially coherent imaging systems, while the underlying assumption of RL-deconvolution is that the imaging system is an incoherent one. It is not clear a priori which OTF must be assumed as valid for GC-RL deconvolution. The deconvolution outcome itself is sensitive to the assumed OTF [5], which motivates the additional question which assumed OTF provides an optimal deconvolution outcome. The third challenge concerns the required upsampling to generate the images at the desired higher resolution. A suitable, computationally efficient method is needed that results in robust and reliable deconvolution outcomes.

In this work we present a comprehensive assessment of the capabilities of GC-RL deconvolution, focusing on the aforementioned challenges. In the Methods section we briefly explain the essentials of the GC-RL algorithm and advances concerning the choice of OTF that is used in the update step and the upsampling of undersampled input data. In the Results section we investigate the algorithm on simulated data, on experimental fluorescence data and on experimental brightfield data of a breast tumour tissue slide. The paper is concluded with a discussion and conclusion.

## 2. Methods

The Richardson-Lucy algorithm can be derived within the framework of Maximum-Likelihood Estimation (MLE), see e.g. Reference [5] for a summary. The image formation can be written as:

(1) μ=g⋅x,

where x corresponds to the ground truth to be estimated, g to the Point Spread Function (PSF) of the microscope, μ to the expected image. Here x and μ are considered as vectors in object and image space, and g as an operator that maps vectors in object space to vectors in image space. The actually observed image is:

(2) M=Poisson(μ),

adding shot noise to the expected image according to Poisson statistics. The RL-update rule for going from iteration n to iteration n+1 is:

(3) $$x^{\left(n+1\right)}=x^{\left(n\right)}\left[g^{T}\cdot \frac{M}{\left(g\cdot x^{\left(n\right)}\right)}\right].$$

Here, it is important that M represents the image data measured in recorded photo-electrons, it is assumed that a gain and offset correction has been applied to the raw image data.

According to the Gradient Consensus (GC) method [14], the measured image M is split into two noise independent halves $M_{1}$ and $M_{2}$ . And according to the random binomial data splitting method [15,22,23], the RL update rule is applied to both data halves independently. Given an iteration n , this results in two predictions $x_{1}^{\left(n+1\right)}$ and $x_{2}^{\left(n+1\right)}$ for iteration n+1 . These define the so-called consensus map:

(4) $$CM=B\cdot \left[\left(x_{1}^{\left(n+1\right)}-x^{\left(n\right)}\right)\left(x_{2}^{\left(n+1\right)}-x^{\left(n\right)}\right)\right],$$

where B is a blurring kernel that is proposed to be $B=g^{†}\cdot g$ [14]. The GC-RL update rule is then defined as:

(5) $$x_{GC}^{\left(n+1\right)}=\left(CM>0\right)x^{\left(n+1\right)}+\left(CM\leq 0\right)x^{\left(n\right)},$$

i.e. the RL-update rule is only applied to the pixels with a positive consensus map, that is for pixels where the RL-update for the two data halves agree if the estimate should increase or decrease (blurred with the kernel B ). In this way over-fitting to noise is prevented, converging to an estimate that balances contrast improvement with noise amplification.

The GC-RL update rule is, strictly speaking, no longer an optimizer for the Poisson statistics based log-likelihood. This calls into question what should be used as stopping criterion for the iterative algorithm. Manton and co-workers [14] propose to use the Kullback–Leibler (KL) divergence (also called relative entropy) [24]. The KL-divergence for the updates of the two data halves is:

(6) $$KL_{1}^{\left(n\right)}=\sum_{k}p_{k}^{\left(n\right)}\log\left(p_{k}^{\left(n\right)}/q_{1,k}\right),$$

(7) $$KL_{2}^{\left(n\right)}=\sum_{k}p_{k}^{\left(n\right)}\log\left(p_{k}^{\left(n\right)}/q_{2,k}\right),$$

where the sum is over pixels k and where $p_{k}^{\left(n\right)}=x_{k}^{\left(n\right)}/\sum_{j}x_{j}^{\left(n\right)}$ , $q_{1,k}=M_{1,k}/\sum_{j}M_{1,j}$ , and $q_{2,k}=M_{2,k}/\sum_{j}M_{2,j}$ are normalized probability distributions associated with the object estimate at iteration n and with the two data halves. The proposed convergence criterion of the deconvolution is that both $KL_{1}^{\left(n+1\right)}$ and $KL_{2}^{\left(n+1\right)}$ have increased compared to $KL_{1}^{\left(n\right)}$ and $KL_{2}^{\left(n\right)}$ , respectively. Other criteria for convergence are quite conceivable, we have decided to simply follow the proposed recipe.

We have added three extensions to the GC-RL method. First, we consider cases where there is also a Gaussian readout noise component (with standard deviation σ ) present. This has consequences for the random binomial data splitting method [15], as well as for the RL-update rule [5]. Second, we explore different forms of the assumed OTF in the deconvolution. The OTF is the Fourier Transform (FT) of the PSF operator g , indicated by a hat as $\hat{g}$ . Specifically we use an OTF as function of spatial frequency q :

(8) $$\hat{g}(q)={\left[{\hat{g}}_{\mathrm{sc}}(\alpha q)\right]}^{\beta },$$

where

(9) $${\hat{g}}_{\mathrm{sc}}(q)=\frac{2}{\pi }\left[\arccos\left(\frac{q\lambda }{2\mathrm{NA}}\right)-\left(\frac{q\lambda }{2\mathrm{NA}}\right)\sqrt{1-{\left(\frac{q\lambda }{2\mathrm{NA}}\right)}^{2}}\right],$$

is the ideal scalar diffraction incoherent OTF, α is a scaling parameter, and β an exponentiation parameter. We use this representation to effectively take into account the impact of several aspects of the optical imaging. First of all, we apply the deconvolution to brightfield microscopy, for which the imaging is partially coherent, which leads to an effective OTF as e.g. derived from an edge response that varies with the partial coherence factor [25]. In addition, there are also differences between the ideal scalar diffraction OTF with a vector diffraction based OTF, which is more realistic for systems with medium to high Numerical Aperture (NA), which are in the range 0.40 to 0.95. Further effects can also arise from unknown optical aberrations. A final effect that may play a role is the camera system used, which can also influence the actual transfer function of the imaging system [26]. Another reason to use the scaling and exponentiation of the OTF is that retrieved contrast and gain in Spectral Signal to Noise Ratio (SSNR) depend on the assumed OTF in the deconvolution [5].

Here, we emphasize that the α and β tuning introduces a controlled model mismatch between the true system transfer function and the empirically adjusted OTF used in deconvolution, while the RL update remains internally matched (using the same $\hat{g}$ in the forward step and its adjoint ${\hat{g}}^{T}$ in the backward step). This differs from unmatched-backprojector acceleration methods (e.g. the WB-ARL method proposed in [27]), where the backprojector is intentionally chosen different from its adjoint ( $B\neq A^{T}$ ) to reshape the forward–backward spectral product and reduce the number of required iterations.

The third extension to the GC-RL method is the generation of an upsampled deconvolution estimate. This means that an object estimate on a K×K grid is desired, whereas the image data is acquired on a N×N pixel grid, with s=K/N>1 the upsampling factor. This can be achieved by upsampling the data by e.g. the standard technique of zero padding in Fourier space followed by filling the extra Fourier space pixels with noise content to maintain Poisson statistics in real space [28]. Here, we describe an alternative method that makes use of downsampling and upsampling during the iterative update process. The mathematical description of the image formation process is now changed to:

(10) μ=D⋅g⋅x,

where D is a downsampling operator from the sN×sN object space to the N×N image space. This changes the PSF to D⋅g , which will result in a modified RL-update rule, consistent with the MLE principle:

(11) $$x^{\left(n+1\right)}=x^{\left(n\right)}\left[g^{T}\cdot U\cdot \frac{M}{\left(D\cdot g\cdot x^{\left(n\right)}\right)}\right],$$

where $U\equiv D^{T}$ is the upsampling operator. The sequence of processing steps now comprises low-pass filtering with the OTF of the estimate at iteration n in object space, then downsampling to image space, computing the pixelwise ratio of image data and expected image, and subsequently upsampling again to object space and low-pass filtering with the transpose of the OTF. This results in the RL-update factor that is used in the pixelwise multiplication to generate the next object estimate at iteration n+1 .

In the following we only consider twofold upsampling ( s=2 ), and simple pixel binning as downsampling. An object space vector x is grouped into blocks of 2×2 pixels, and for each block we take the sum over the 2×2 pixel values. This results in the downsampled image space vector D⋅x . The corresponding upsampling operator then takes each pixel of an image space vector y and creates 2×2 copies. This results in the upsampled object space vector U⋅y . Mathematically:

(12) $${\left(D\cdot x\right)}_{ij}=\sum_{p=0}^{1}\sum_{q=0}^{1}x_{2i+p,2j+q},$$

(13) $${\left(U\cdot y\right)}_{2i+p,2j+q}=y_{ij},\;p,q\in \left\{0,1\right\},$$

where we use a notation with two integers labelling the row and column position on the pixel grid. Note that D and U are non-square so that D⋅U , operating in image vector space, is equal to (4×) the unity operator, but U⋅D , operating in object vector space, is not.

## 3. Results

### 3.1. Deconvolution of Siemens star simulation data

We simulated a sinusoidal Siemens star object with 100 cycles with 513×513 pixels (pixel size regarded as 1 µm). The object was convolved with the ideal scalar diffraction OTF with cutoff spatial frequency at $1/\sqrt{2}$ of the sampling rate, i.e. at half of the maximum spatial frequency along the diagonal in Fourier space, i.e. the object and image are slightly oversampled. Then, shot noise was added according to the Poisson distribution taking an average of 1000 photons per pixel, and the GC-RL algorithm was used to reconstruct the image with the same OTF. This was repeated 10 times, resulting in 10 noise-independent reconstructions. The mean and standard deviation over all 10 reconstructions was computed, as well as the power spectrum (mean of absolute value squared) and noise variance in Fourier space. In order to enable tracking the development of Spectral Signal to Noise Ratio (SSNR) as a function of iteration number we kept the number of iterations constant, since the SSNR is computed from multiple (ten) noise-independent realizations and a meaningful comparison requires the iteration number to be identical for all cases. The automatic stopping criterion gave rise to a number of iterations in the range of about 40 to 50, so we have set the constant number of RL iterations equal to 50.

**Fig. 1.**
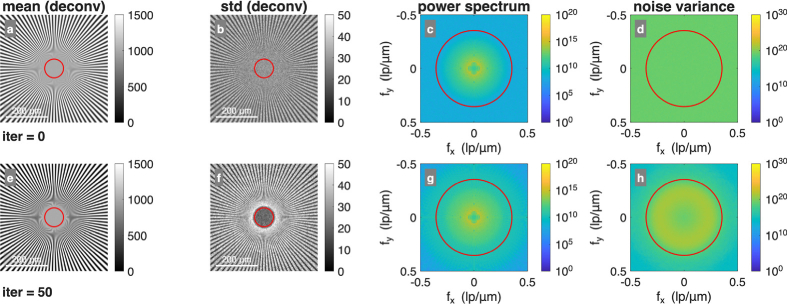
Outcome of GC-RL deconvolution on simulated Siemens star object. Image and Fourier statistics before (panels a,b,c,d) and after (panels e,f,g,h) iterations. (a,e) image mean, (b,f) image standard deviation, (c,g) Fourier squared mean, (d,h) and Fourier variance. The red circle line implies the diffraction limit of the OTF, i.e. the area where the pitch of the spokes is less than λ/2NA (panels a,b,e,f) or where the spatial frequency is less than 2NA/λ (panels c,d,g,h).

Figure 1 shows the outcome of the simulation. The contrast of the reconstruction is improved compared to the input without a large increase in the standard deviation, in agreement with the key strength of the GC-RL method. The improvement does not extend to the inner zone, where the periodicity is below the diffraction limit. This matches the characteristic that any RL-deconvolution enhances point and edge features, but not periodic objects. In Fourier space, we observe an increase in the content at higher spatial frequencies and a slightly enhanced noise level for spatial frequencies just below the diffraction limit.

**Fig. 2.**
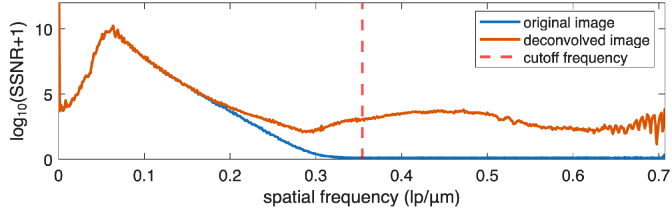
Ring-averaged SSNR before and after GC-RL deconvolution on simulated Siemens star object.

Visualization [29] shows the development of Spectral Signal to Noise Ratio (SSNR), averaged over rings in Fourier space, as the iterative process of GC-RL deconvolution progresses. Figure 2 shows the initial and final SSNR. Initially, the SSNR exhibits the largest increase near the cutoff frequency region. As iterations proceed, nearby frequency components are gradually recovered, eventually reaching a comparable level. However, after approximately 10 iterations, a decline in SSNR is observed in certain regions, which corresponds to the onset of noise amplification where the Fourier variance begins to grow faster than the Fourier squared mean. It appears that noise amplification is strongly reduced but not fully eliminated.

**Fig. 3.**
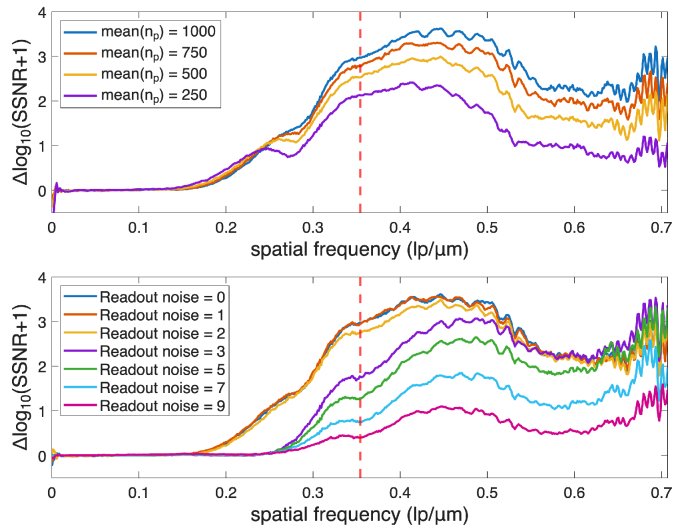
Ring-averaged SSNR improvement on simulated Siemens star object regarded as different SNR of the captured image (top) and different level of readout noise of the sensor (bottom). The red dashed line implies the diffraction limit of the OTF.

We evaluated the information recovery ability of the GC-RL algorithm as a function of noise level. To that end, we repeated the simulation process by reducing the average number of photons per pixel from 1000 to 250. In addition, we ran simulations where Gaussian readout noise was added on top of the shot noise. As described in Section 2, a modified image-splitting strategy and update rule were applied to approximately preserve Poisson noise statistics, thereby mitigating the influence of readout noise. Figure 3 shows the final ring averaged SSNR as a function of spatial frequency for the different signal and readout noise levels. It appears that a stronger increase in SSNR levels is generated by the GC-RL algorithm in case the noise level of the raw input images is weaker. Signal levels of more than roughly 100 photons per pixel on average are needed for a sizeable increase in SSNR, and readout noise levels of more than a handful of photo-electrons per pixel stand in the way of successful enhancement of SSNR by GC-RL deconvolution.

Intuitively, one might expect that taking the true OTF as the assumed OTF provides the optimal basis for deconvolution, as it represents the true spatial frequency response of the imaging system. It turns out in practice, however, that some modifications to the OTF can contribute to improved reconstruction results. We simulated GC-RL deconvolution outcomes for different values of the OTF scaling parameter α and OTF exponentiation parameter β , as defined in Section 2.

**Fig. 4.**
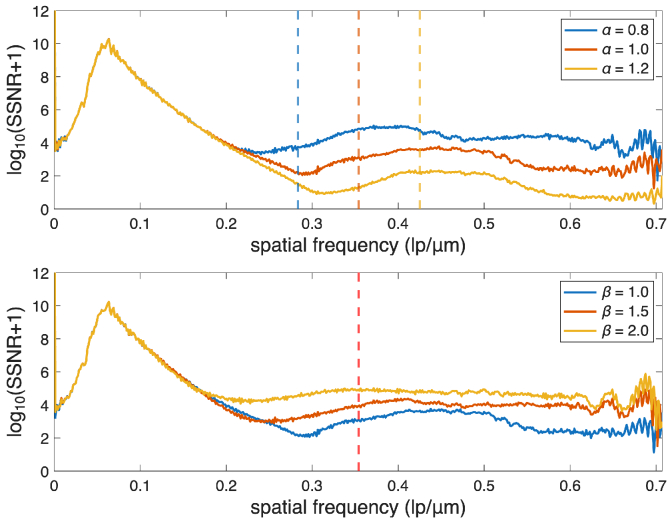
Ring-averaged SSNR improvements for a simulated Siemens star object under different OTF modification schemes. The top panel shows results obtained using OTF stretching with scaling parameter α , while the bottom panel shows results obtained using OTF powering with exponentiation parameter β . The three dashed lines indicate the diffraction limits of the modified OTFs, corresponding to α=0.8 (blue), α=1.0 (red), and α=1.2 (yellow).

We compared the nominal case in which the assumed OTF in the deconvolution is the same as the ground truth OTF in the forward model ( α=1 ) to the case with a deconvolution OTF with reduced cutoff frequency at 80% (squeezed OTF, α=0.8 ), and with a deconvolution OTF with extended cutoff frequency at 120% (stretched OTF, α=1.2 ). Figure 4 shows that GC-RL deconvolution yields higher SSNR gains when a relatively narrower deconvolution OTF is used (i.e., retaining around 80–90% of the original OTF bandwidth). However, further reducing the cutoff below this range is not recommended, as excessive OTF compression significantly attenuates high-frequency components, limiting the maximum recoverable spatial frequency. In practice, a moderately reduced OTF ( α≈0.8 – 0.9 ) generally provides a good balance between SSNR enhancement and spatial resolution.

Next, we performed GC-RL deconvolutions for different OTF exponentiation parameters β . Figure 4 shows that the SSNR gain increases with β , which appears to be the result of edge enhancement. A practical trade-off of OTF modification is that strong exponentiation can alter the appearance of periodic structures, transforming smooth sinusoidal patterns into more block-like profiles. Excessive OTF exponentiation may even introduce edge overshoot and ringing artifacts, which degrades the quality of the deconvolution results. We found that a visually optimum balance of this effect with contrast enhancement and information restoration is approximately achieved at a power of β=1.5 . In addition to these visual effects, the OTF parameters also influence the convergence behavior when an automatic stopping criterion is used. Using the KL-divergence-based stopping in additional Siemens-star simulations, we observed that moderate variations of the OTF parameters ( α approximately 0.9–1.0 and β approximately 1.00–1.25) result in stopping after a comparable number of iterations. In contrast, more pronounced OTF modifications, specifically smaller α or larger β , systematically increase the number of iterations required before the KL-based stopping condition is met.

**Fig. 5.**
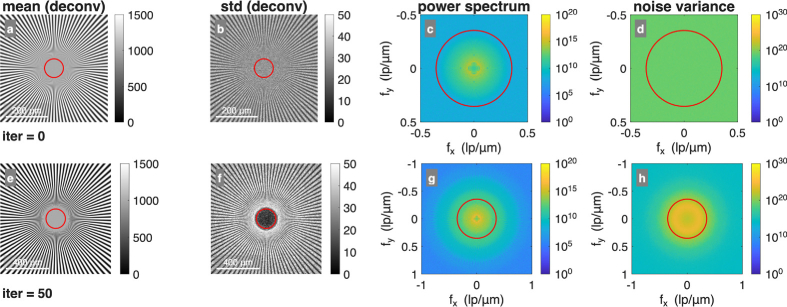
Outcome of GC-RL deconvolution on simulated Siemens star object. Image and Fourier statistics before (panels a,b,c,d) and after (panels e,f,g,h) iterations. (a,e) image mean, (b,f) image standard deviation, (c,g) Fourier squared mean, (d,h) and Fourier variance. The red circle line implies the diffraction limit of the OTF, i.e. the area where the pitch of the spokes is less than λ/2NA (panels a,b,e,f) or where the spatial frequency is less than 2NA/λ (panels c,d,g,h). The cutoff frequency value remain the same in (g,h), but the highest frequency has been doubled as a result of upsampling.

**Fig. 6.**
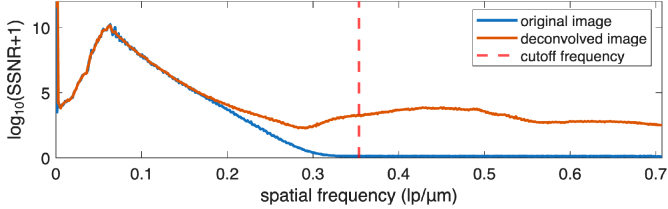
Ring-averaged SSNR before and after deconvolution. Only the original frequency range is shown here for a better illustration of the SSNR improvement around the diffraction limit.

In a next step we tested the upsampling-embedded version of the GC-RL deconvolution using the downsampling operator D (2×2 pixel binning to the lower sampling rate in image space) and its adjoint operator, the upsampling operator U (2×2 pixel copying to the higher sampling rate in object space). In this way deconvolution and upsampling are jointly performed. Figure 5 shows the mean and spread of the input raw images and deconvolution outcome in real and Fourier space. We see a similar improvement in image contrast without large noise enhancement, just as for the case without upsampling embedding shown in Fig. 1. The same conclusion applies to the SSNR gain shown in Fig. 6, which is very similar to the result shown in Fig. 2.

### 3.2. Deconvolution of fluorescence microscopy experimental data

**Fig. 7.**
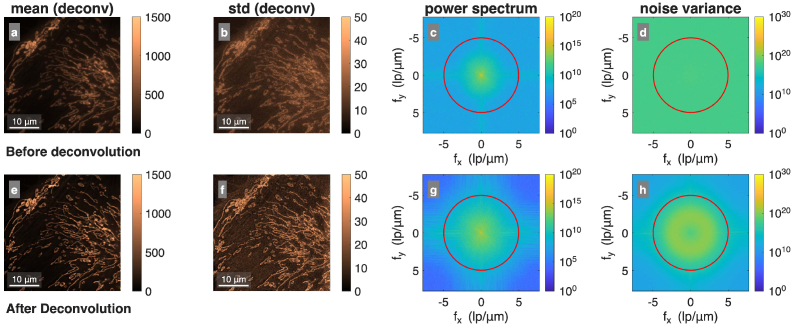
Outcome of GC-RL deconvolution on experimental fluorescence microscopy data. Image and Fourier statistics before (panels a,b,c,d) and after (panels e,f,g,h) iterations. (a,e) image mean, (b,f) image standard deviation, (c,g) Fourier squared mean, (d,h) and Fourier variance. The red circle line implies the diffraction limit of the OTF, i.e. the area where the spatial frequency is less than 2NA/λ (panels c,d,g,h).

We tested the GC-RL algorithm on experimental fluorescence microscopy data used by us before [5]. In short, a sample of Bovine Pulmonary Arterial Endothelial (BPAE) cells was imaged with different magnification and Numerical Aperture (NA) values, using a monochrome sCMOS sensor (3200×3200 pixels, pixel size 6.5 µm). Here, we have only looked at the SSNR of the final outcome of the deconvolution, implying that a variable number of RL iterations for the different noise realizations when using the automated stopping criterion is not a problem. Figure 7 shows the GC-RL deconvolution result for the 100×/NA1.49 objective data (mitochondria, red channel). Clearly, image contrast and SNR are dramatically enhanced without large noise enhancement, in agreement with the outcomes of the Siemens star simulations.

**Fig. 8.**
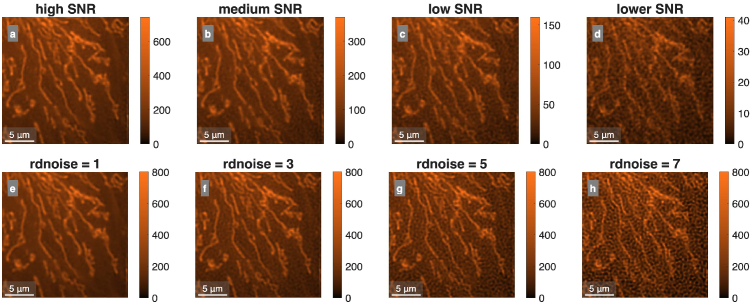
Zoomed-in deconvolution outcome of the captured experimental fluorescence microscopy image with different SNR of the exposure time (a,b,c,d) or with different level of readout noise of the sensor (e,f,g,h).

**Fig. 9.**
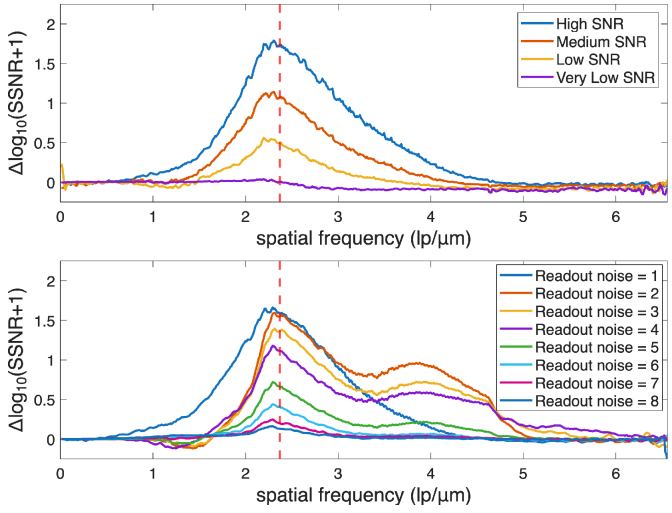
Ring-averaged SSNR improvement on experimental fluorescence microscopy data regarded as different SNR of the captured image (top) and different level of readout noise of the sensor (bottom). The red dashed line implies the diffraction limit of the OTF.

In a next step we analysed data acquired with a 40×/NA0.75 objective with an extra 1.5× magnification adapter with different exposure times of the camera, thus providing a range of signal strength levels. We also added Gaussian noise at a range of different root mean square values to emulate the effect of using a camera with higher readout noise than the sCMOS one that was actually used (readout noise around 1 photon-electron per pixel according to specifications). To maximize the deconvolution SSNR improvement, the OTF has been stretched ( α=0.75 ) for the 40×/NA0.75 setup to match the spatial frequency where the spectral content of the image approaches the noise floor. Figure 8 shows the outcome of the GC-RL deconvolution for the different cases, Fig. 9 shows the gain in the ring averaged SSNR. The gain in SSNR peaks at the cutoff spatial frequency of the stretched OTF ( α=0.75 ) used in the deconvolution, and this peak increases with input signal level. When this drops to values well below 100 photons per pixel the gain in SSNR by GC-RL deconvolution vanishes. Similarly, for added Gaussian noise, emulating readout noise effects, the peak in SSNR gain decreases, dropping to near zero for readout noise levels approaching 10 photo-electrons per pixel. These results agree with the findings of the Siemens star simulation study. One notable difference is that, at higher readout noise levels, the SSNR in the high-frequency regions has better recovery results than for the lower readout noise conditions.

**Fig. 10.**
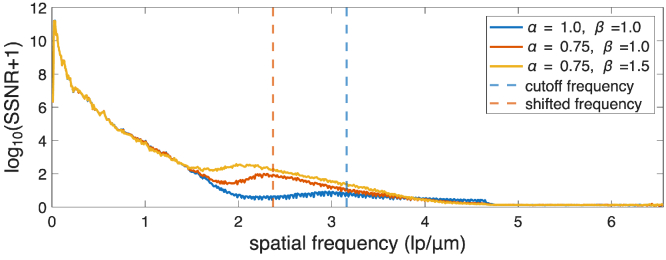
Ring-averaged SSNR results after deconvolution using different OTF design on experimental fluorescence microscopy data.

As illustrated in Fig. 10, the recovered SSNR values are improved by stretching the OTF to align with the spatial frequency where the spectral content of the image approaches the noise floor ( α=0.75 ). The red dashed line here indicates the cutoff of the stretched deconvolution OTF. In addition, applying a 1.5-powered OTF ( β=1.5 ) can further enhance image contrast and sharpness, result in better improved visual clarity in the reconstructed image.

### 3.3. Deconvolution of brightfield microscopy experimental data

As a final test case we imaged Haematoxylin and Eosin (H&E) stained human breast carcinoma tissue sections with a 10×/NA0.45 objective lens with brightfield illumination. Full-colour imaging was achieved by sequential acquisition using red, green, and blue filters (FF01-676/29, FF01-545/55, and FF01-460/60; from Semrock).

The GC-RL deconvolution algorithm was applied to all three image sets and then calibrated to generate a full colour RGB image. Since the transmittance and the bandwidth of the three colour filters differs, the white-illumination background is used as the calibration reference. The scaling factors for the three colour channels are calculated by matching both the colour balance and the maximum brightness. The resulting RGB colour correction factors for 10× and 20× magnification were found to be [1.11; 1.07; 1.08] and [1.70; 1.30; 1.31], respectively.

We used the upsampling-embedded version of the GC-RL algorithm to double the sampling rate. The reason is that the system is sampling-limited (back projected pixel size of raw acquisitions is 0.65 μ m) rather than diffraction-limited (Nyquist sampling distance λ/(4NA)≈0.3μm for the green channel). The imaging and subsequent deconvolution was repeated 10× to measure the mean and variance in real and Fourier space as well as ring averaged SSNR curves.

**Fig. 11.**
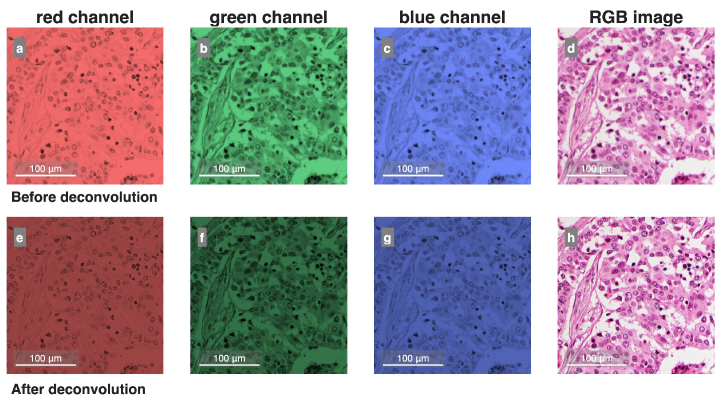
Raw images (a,b,c,d) and deconvolved images (e,f,g,h) acquired with red (a,e), green (b,f), and blue (c,g) bandpass filters, and the calibrated merged RGB image (d,h) of experimental brightfield microscopy data.

**Fig. 12.**
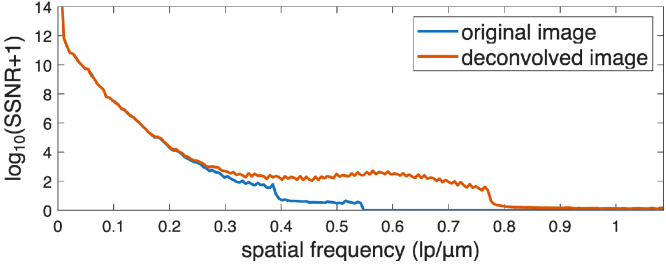
Ring-averaged SSNR before and after deconvolution for brightfield green channel of the experimental brightfield microscopy data.

The primary results are shown in Fig. 11 and Fig. 12. The GC-RL deconvolution provides a clear enhancement of structure, as the nucleoli features become sharper and the intracellular texture is more clearly resolved. The SSNR is mainly improved for the higher spatial frequencies, and not so much for the spatial frequencies just below the original sampling rate, which we attribute to the undersampling of the acquired data. Note that we only show the SSNR result for the green channel, as the results for the red and blue channel were nearly identical.

**Fig. 13.**
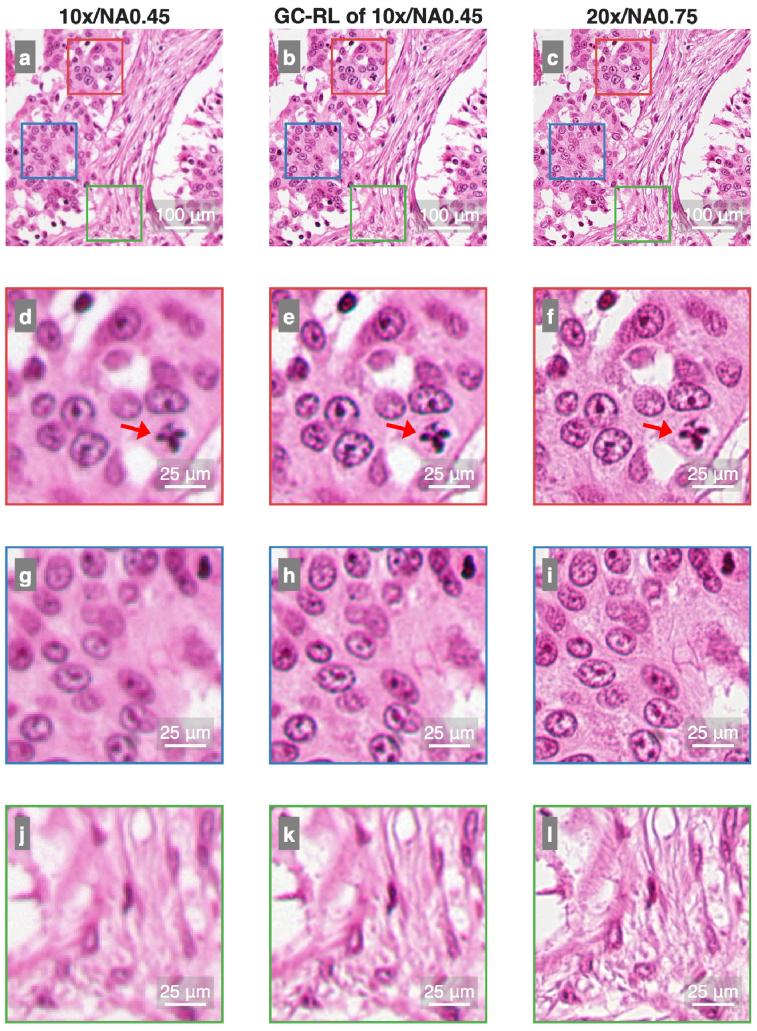
Image quality comparison of (a,d,g,j) 10×/0.45 NA original images, (b,e,h,k) 10×/0.45 NA upsampling-embedded deconvolved images, and (c,f,i,l) 20×/0.75 NA original images. Panels (a–c) show 800×800 pixel regions, while (d–f), (g–i), and (j–l) are corresponding 200×200 pixel zoomed-in views highlighting fine structural details. Representative high-frequency nuclear structures (red arrows), including mitosis-like figures, become more discernible after GC-RL reconstruction, approaching the visual clarity of images acquired with a 20×/NA0.75 objective.

**Fig. 14.**
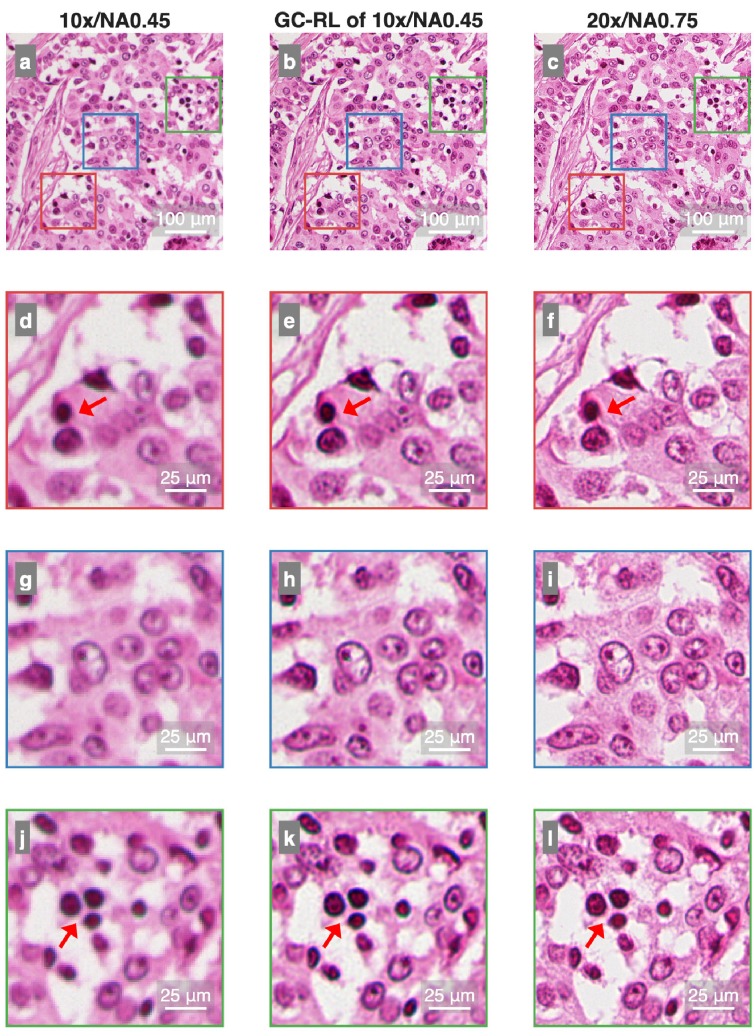
Image quality comparison of (a,d,g,j) 10×/0.45NA original images, (b,e,h,k) 10×/0.45NA upsampling-embedded deconvolved images, and (c,f,i,l) 20×/0.75NA original images. Panels (a–c) show 800×800 pixel regions, while (d–f), (g–i), and (j–l) are corresponding 200×200 pixel zoomed-in views highlighting fine structural details. Representative high-frequency nuclear structures (red arrows), including irregular nucleoli figures, become more discernible after GC-RL reconstruction, approaching the visual clarity of images acquired with a 20×/NA0.75 objective.

We compared the outcome of the GC-RL deconvolutions also to undeconvolved data acquired with a 20×/NA0.75 objective (see Fig. 13 and Fig. 14). In both figures, the upsampled GC-RL reconstructions shows consistently higher visual resolution than the native 10×/NA0.45 raw image. Cell boundaries become more delineated, nucleoli are more identifiable and intranuclear texture appears with greater local contrast. These observed enhancements directly improve the visibility of these features relevant to pathology diagnosis. This diagnostic assessment relies heavily on nuclear size and shape, chromatin texture, nuclear-to-cytoplasmic ratio, and the presence of so-called mitotic figures [20,21]. The upsampled GC-RL makes these features more visible by recovering additional higher frequency structural details, while avoiding common deconvolution artifacts such as strong ringing and noise amplification. Notwithstanding the clear improvement over the native 10×/NA0.45 data, the GC-RL deconvolution outcome does not reach the full potential of the true higher resolution imaging of the 20×/NA0.75 objective. Specifically, smaller features with high intrinsic contrast like the nucleoli are recovered relatively well, while lower contrast textures in a relatively uniform background are not that well retrieved.

## 4. Conclusion

In this study , we have presented an evaluation of practical aspects of the Gradient-Consensus Richardson-Lucy (GC-RL) deconvolution algorithm for microscopy image deconvolution. GC-RL enhances image contrast and structural detail while suppressing noise amplification, and demonstrates robust performance across simulated and experimental datasets under realistic microscopy conditions.

**Fig. 15.**
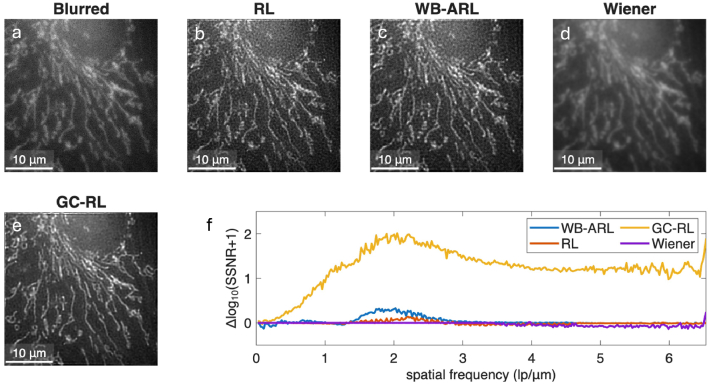
Comparison of image restoration methods on fluorescence microscopy data (a) and deconvolution results obtained using (b) conventional Richardson–Lucy (RL), (c) Wiener–Butterworth accelerated RL (WB-ARL), (d) Wiener deconvolution, and (e) Gradient-Consensus RL (GC-RL). (f) Ring-averaged SSNR improvement for the different deconvolution methods.

We compared the GC-RL to a limited set of other image restoration and deconvolution methods on the fluorescence data of section 3.2. An exhaustive comparison to a broad range of methods for different samples over a range of different experimental conditions is outside the scope of the current study. Figure 15(a–e) shows the outcome of the comparison of the GC-RL method to conventional RL deconvolution, Wiener–Butterworth accelerated RL deconvolution [27], and Wiener deconvolution. The GC-RL algorithm yields a visibly cleaner background while preserving fine structural details, whereas the other methods either suffer from noise-related artifacts or produce more conservative reconstructions. The ring-averaged SSNR analysis in Fig. 15(f) is consistent with these observations, showing a clear SSNR improvement for GC-RL over a broad spatial frequency range compared to the other algorithms.

In practice, the effectiveness of GC-RL deconvolution depends on the noise conditions of the input data. The deconvolution gain, quantified by the difference in SSNR between the deconvolution outcome and the input, increases with the SNR level of the input data. For signal levels below about 100 photons per pixel and readout noise levels above about 5 photo-electrons per pixel, the gain approaches zero, implying that GC-RL deconvolution cannot be successfully applied to data with a very high noise level. Robustness, however, is found for application to data that does not strictly follow incoherent image formation such as brightfield microscopy data. In experimental microscopy settings, it is worth noting that modern sCMOS cameras typically exhibit readout noise well below this threshold, whereas legacy clinical scanners utilizing traditional CCD sensors may exceed it, which should be taken into account when considering deployment in existing clinical infrastructure.

Tailoring the assumed OTF in the deconvolution is a powerful tool to optimize the GC-RL deconvolution results. A deconvolution OTF, effectively parameterized with a scaling parameter and an exponentiation parameter, improves the image contrast and SSNR gain that can be achieved with the deconvolution. Embedding the upsampling operation in the iterative steps provides a simple, yet crucial element to applying deconvolution to undersampled data, providing a good reconstruction on the upsampled pixel grid.

A subtle consequence of the suppressed noise enhancement of the gradient-consensus mechanism is the dependence of contrast enhancement on the inherent contrast of the sample. The GC-RL update is conservative for very low-contrast textures under noisy conditions, because updates are gated by agreement between the two noise-independent splits. Features with relatively low contrast are under-recovered as they approach the noise level. We have verified this effect with additional Siemens-star simulations with varying intrinsic contrast. These simulations indicate that the SSNR gain decreases with intrinsic contrast and becomes negligible when the modulation contrast drops to approximately 40% for the noise levels considered. GC-RL is therefore most effective when relevant features have sufficient intrinsic contrast.

These findings may have great practical application potential in the field of Whole-Slide-Imaging (WSI) for Digital Pathology. The recovery of higher-resolution details from lower-magnification images without prior assumptions offers the benefits of a major step in scanning speed (measured in area/time), and in the attendant data storage. Specifically, key high-resolution features of diagnostic relevance such as mitotic figures could be retrieved with good quality from 10×/NA0.45 data, obviating the need to acquire full data of entire tissue slides with 20×/NA0.75 objectives.

In future work, further efforts will be focused on improving the computational efficiency of the modified GC-RL framework to enable large-scale applications. Our current Matlab implementation on megapixel-scale patches indicates that the per-iteration cost of GC-RL can be more than an order of magnitude higher than conventional RL, motivating dedicated acceleration and implementation work. Acceleration strategies such as Wiener–Butterworth unmatched-backprojector acceleration [27], vector-based Biggs-Andrews acceleration [30,31], and GPU-based implementations [32,33] could substantially reduce processing time.

Another important direction would be the extension of the modified GC-RL framework to three-dimensional deconvolution. In current brightfield microscopy applications, the lack of true optical sectioning leads to reduced axial contrast and strong out-of-focus background. Extending the framework to 3D, potentially combined with tailored OTF models, may provide further contrast enhancement and resolution recovery.

## Data Availability

Fluorescence microscopy data is available at [34]. The used Matlab code for GC-RL deconvolution is available at [29]. Brightfield microscopy data is provided as courtesy by Arno van Leenders and is not publicly available.
